# Comparison between Enteroscopy-, Laparoscopy- and Endoscopic Ultrasound-Assisted Endoscopic Retrograde Cholangio-Pancreatography in Patients with Surgically Altered Anatomy: A Systematic Review and Meta-Analysis

**DOI:** 10.3390/life12101646

**Published:** 2022-10-20

**Authors:** Paraskevas Gkolfakis, Apostolis Papaefthymiou, Antonio Facciorusso, Georgios Tziatzios, Daryl Ramai, Spyridon Dritsas, Theodosia Florou, Ioannis S. Papanikolaou, Cesare Hassan, Alessandro Repici, Konstantinos Triantafyllou, Lars Aabakken, Jacques Devière, Torsten Beyna, Marianna Arvanitakis

**Affiliations:** 1 Department of Gastroenterology, Hepatopancreatology, and Digestive Oncology, CUB Erasme Hospital, Université Libre de Bruxelles (ULB), 1070 Brussels, Belgium; 2 Pancreaticobiliary Unit, University College London Hospitals, London NW1 2BU, UK; 3 Gastroenterology Unit, Department of Surgical and Medical Sciences, University of Foggia, 71122 Foggia, Italy; 4 Hepatogastroenterology Unit, Second Department of Internal Medicine-Propaedeutic, Medical School, National and Kapodistrian University of Athens, “Attikon” University General Hospital, 124 62 Chaidari, Greece; 5 Gastroenterology and Hepatology, University of Utah Health, Salt Lake City, UT 84132, USA; 6 Department of General Surgery, Yeovil District Hospital NHS Foundation Trust, Higher Kingston, Yeovil BA21 4AT, UK; 7 Department of Gastroenterology, University Hospital of Larissa, 411 10 Larissa, Greece; 8 Endoscopic Unit, Department of Gastroenterology, IRCCS Humanitas Research Hospital, 20089 Milan, Italy; 9 GI Endoscopy Unit, Institute of Clinical Medicine, Oslo University Hospital, 0372 Oslo, Norway; 10 Department of Internal Medicine, Evagelisches Krankenhaus Düsseldorf, 40225 Düsseldorf, Germany

**Keywords:** ERCP, Billroth, Roux-n-Y, RYGB, EDGE, enteroscopy, balloon enteroscopy, laparoscopy

## Abstract

Background and Aims: Endoscopic retrograde cholangiopancreatography (ERCP), in surgically altered anatomy (SAA), can be challenging and the optimal technique selection remains debatable. Most common foregut interventions resulting to this burden consist of Billroth II gastrectomy, Whipple surgery and Roux-en-Y anastomoses, including gastric by-pass. This systematic review, with meta-analysis, aimed to compare the rates of successful enteroscope-assisted (EA)-, endosonography-directed transgastric- (EDGE), and laparoscopy-assisted (LA)-ERCP. Methods: A systematic research (Medline) was performed for relative studies, through January 2022. The primary outcome was technical success, defined as approaching the ampulla site. Secondary outcomes included the desired duct cannulation, successful therapeutic manipulations, and complication rates. We performed meta-analyses of pooled data, and subgroup analysis considering the EA-ERCP subtypes (spiral-, double and single balloon-enteroscope). Pooled rates are reported as percentages with 95% Confidence Intervals (95%CIs). Results: Seventy-six studies were included (3569 procedures). Regarding primary outcome, EA-ERCP was the least effective [87.3% (95%CI: 85.3–89.4); I^2^: 91.0%], whereas EDGE and LA-ERCP succeeded in 97.9% (95%CI: 96.4–99.4; I^2^: 0%) and 99.1% (95%CI: 98.6–99.7; I^2^: 0%), respectively. Similarly, duct cannulation and therapeutic success rates were 74.7% (95%CI: 71.3–78.0; I^2^: 86.9%) and 69.1% (95%CI: 65.3–72.9; I^2^: 91.8%) after EA-ERCP, 98% (95%CI: 96.5–99.6; I^2^: 0%) and 97.9% (95%CI: 96.3–99.4) after EDGE, and 98.6% (95%CI: 97.9–99.2; I^2^: 0%) and 98.5% (95%CI: 97.8–99.2; I^2^: 0%) after LA-ERCP, respectively. The noticed high heterogeneity in EA-ERCP results probably reflects the larger number of included studies, the different enteroscopy modalities and the variety of surgical interventions. Comparisons revealed the superiority of LA-ERCP and EDGE over EA-ERCP (*p* ≤ 0.001) for all success-related outcomes, though LA-ERCP and EDGE were comparable (*p* ≥ 0.43). ERCP with spiral-enteroscope was inferior to balloon-enteroscope, while the type of the balloon-enteroscope did not affect the results. Most adverse events were recorded after LA-ERCP [15.1% (95%CI: 9.40–20.8); I^2^: 87.1%], and EDGE [13.1% (95%CI: 7.50–18.8); I^2^: 48.2%], significantly differing from EA-ERCP [5.7% (95%CI: 4.50–6.80); *p* ≤ 0.04; I^2^: 64.2%]. Conclusions: LA-ERCP and EDGE were associated with higher technical, cannulation, and therapeutic success compared to EA-ERCP, though accompanied with more adverse events.

## 1. Introduction

Endoscopic retrograde cholangiopancreatography (ERCP) represents a frequent challenge among patients with foregut surgically altered anatomy (SAA). The segmental or complete resection of gastric lumen, especially when accompanied by vagus nerve resection, as well as the abrupt weight loss after bariatric intervention comprise independent risk factors for gallstone disease [1,2,3,4] Moreover, when the indication for surgery is a pancreato-biliary tumor, there is a persistent risk of local recurrence that could necessitate endoscopic reintervention to provide biliary decompression [5].

There is a broad range of surgical interventions on the upper gastrointestinal tract that can influence ERCP success. Cases with Billroth I gastrectomy are currently uncommon, and usually do not impact the ERCP process [6,7] On the other hand, ERCP in patients with previous Billroth II surgery can present difficulty considering the recognition and intubation of the afferent loop with a duodenoscope and the reverse axis of common bile duct (CBD) cannulation and sphincterotomy [8,9,10]. This complexity increases when gastrectomy is combined with Roux-n-Y (RY) anastomosis, where the length of the roux limb adds a barrier to approach the biliopancreatic limb and ampulla using a duodenoscope [11,12,13] Moreover, this anatomical modification represents the vast majority of SAA cases, as it has been adopted to accompany bariatric interventions, and specifically Roux-n-Y gastric bypass (RYGB). Finally, pancreaticoduodenectomy, including Whipple and its variants, share the aforementioned obstacles in ERCP and frequent need for endoscopic reintervention, especially considering the low rates of curative resection and the potential necessity for metachronous CBD drainage [14].

Currently, there is no definite recommendation regarding successful ERCP in patients with SAA. Conventional endoscopes, namely duodenoscopes, gastroscopes and colonoscopes have been proven suboptimal for ductal cannulation, particularly due to their inability to reach the ampulla or the anastomosis [10,15,16] More specifically, the conventional side-viewing duodenoscope, although represents an acceptable choice after Billroth II gastrectomies (62.5%- 86.1%), the success rate in approaching the ampulla reduces dramatically after more complex surgeries, such as RY (75.3%) or Whipple (57.9%) [15,17]. Further modifications, including the use of attachments, guidewires, dilatation balloons, or fluoroscopy guidance have provided positive outcomes in isolated cases, but remain inferior to advanced endoscopic techniques [9,18,19,20]. Among these modalities, balloon assisted-ERCP (BA-ERCP) has been broadly studied, while first generation spiral enteroscopy (manual) has also been evaluated in the past [21,22]. On the other hand, the option to bypass the long way to the ampulla has been facilitated by the implementation of hybrid methods, combining endoscopic ultrasound (EUS) or laparoscopy and ERCP. In this regard, EUS-directed transgastric ERCP (EDGE) and laparoscopy-assisted ERCP (LA-ERCP) aim to ensure direct access to the afferent loop, mainly in RYGB cases. Nevertheless, the vast majority of the current literature on this topic consists of retrospective case series or cohort studies, while randomized controlled trials are absent on this issue. Moreover, relative meta-analyses focused on some of the available modalities applied in certain subgroups, mainly concerning the surgery type, and no comparative data exist assessing all advanced ERCP techniques in SAA cases, regardless of the type of surgery [21,22,23,24,25,26,27] For example, there are comparisons of those modalities in isolated RYGB cases or evaluation of EA-ERCP in all surgery types, but a cumulative assessment of the available advanced techniques in all kinds of surgeries has never been presented. Although the inclusion of studies with a variety of previous surgeries increases the risk of heterogeneity, the applicability of the results regardless the type of surgery could be beneficial in clinical practice.

This systematic review and meta-analysis aimed to evaluate and compare all available advanced ERCP alternatives in patients with SAA and provide results considering the successful positioning in front of the ampulla or the anastomosis, ductal cannulation, and therapeutic success. Based on the main technical characteristics of each procedure, we hypothesized that EDGE could be superior to the other techniques considering success and safety, followed by LA-ERCP for safety reasons and EA-ERCP regarding efficacy.

## 2. Materials and Methods

This study was conducted following a pre-defined registered protocol (PROSPERO Registration Number: CRD42022320978) and its interpretation was in accordance with the Preferred Reporting Items for Systematic Reviews and Meta-Analyses (PRISMA) checklist (Supplementary Table S2) [28].

### 2.1. Inclusion and Exclusion Criteria

The question of our review was addressed based on the PICO framework and included the comparison of the pooled data among advanced ERCP techniques regarding their technical and clinical success [29]. Case-series or cohorts evaluating at least one technique were included in the final analysis when all of the following prerequisites were met: (A) Patients: adult patients, with a history of surgically altered anatomy undergoing ERCP, using (B) Interventions: enteroscope-assisted (EA-) ERCP (single- or double-balloon scope or spiral enteroscope), EDGE, or LA-ERCP (C) Comparators: pooled data of all modalities were compared and reported (D) Outcomes: technical success defined as sufficient scope positioning in front of the ampulla or the hepaticojejunal and/or pancreatico-jejunal anastomosis, rate of deep cannulation of the desired duct and the therapeutic yield of the procedure.

Studies with less than 10 patients per modality or those using conventional endoscopes were excluded. Moreover, alternative modalities to ERCP, such as EUS anterograde cholangiopancreatography, or percutaneous transhepatic cholangiopancreatography were also excluded.

### 2.2. Search Strategy

An initial online search was confined to English language literature on PubMed/Medline and Cochrane library published through 20 January 2022. The search algorithm included the following Boolean search terms: *ERCP* AND (“*altered anatomy*” OR “*Billroth*” OR “*Roux-en-Y*” OR “*Whipple*” OR “*hepaticojejunal*” OR “*EDGE*” OR “*double balloon*” OR “*single balloon*” OR “*spiral scope*”). Additional relevant articles were hand-searched in the reference lists of the retrieved publications and further key review articles, as well as by using the “similar article” function of PubMed. Unpublished works, abstracts, and oral or poster presentations were excluded. In case of missing data, the first and/or the corresponding authors were contacted. Two investigators (AP, PG) independently selected articles of interest based on the aforementioned inclusion and exclusion criteria. In cases of multiple publications from the same study, only the most recent and complete article was included. The retrieved studies all were inserted and managed by the reference manager Mendeley Desktop for Windows v. 1.19.1 (Mendeley Ltd., Elsevier, Amsterdam, The Netherlands).

### 2.3. Data Abstraction and Quality Assessment

Data on study-, participant-, and intervention-related characteristics were abstracted into a standardized form by two investigators (PG, AP) independently; discrepancies were resolved by consensus, referring back to the original article, in consultation with a third reviewer (GT). The quality of the included studies was assessed by two authors independently (AP, GT) using the National Heart, Lung, and Blood Institute (NHLBI) tool for case-series, that allows the evaluation of cohort studies without a comparator [30].

### 2.4. Outcomes

The primary outcome of our meta-analysis was a technical success, as it is the first obstacle to achieve ERCP success in such cases and the main primary outcome of the relative studies and was defined as sufficient positioning of the endoscope tip in front of the ampulla or the anastomosis. Secondary outcomes included the successful desired duct cannulation and the therapeutic yield of the procedure, considered as deep ductal guidewire insertion or opacification and accomplishment of the indicated treatment plan, respectively. Potential adverse events were compared in total and after classification into post-ERCP pancreatitis, cholangitis, bleeding, and perforation cases. Considering EA-assisted-ERCP, further subgroup analysis was performed to compare single- or double-balloon enteroscopy versus first-generation manual spiral enteroscopy.

### 2.5. Statistical Analysis

Study outcomes were pooled through a random-effects model based on the DerSimonian and Laird test, and results are expressed as rates and 95% confidence intervals (CI). The presence of heterogeneity was calculated through I^2^ tests with I^2^ < 20% interpreted as low-level and I^2^ between 20% to 50% as moderate heterogeneity. The pooled outcome rates of the different techniques were compared using the bivariate approach [31]. Publication bias assessment was performed at least with visual evaluation of funnel plots. The analyses were performed using Comprehensive Meta-Analysis software, version 3 (BioStat, Englewood, NJ, USA). For all calculations a two-tailed *p* value of less than 0.05 was considered statistically significant.

### 2.6. Quality of Evidence

GRADE criteria were used to rate the quality of evidence derived from the meta-analysis. Using this approach, RCTs are considered to have the highest quality of evidence and can be down rated based on bias, imprecision, or heterogeneity in the data. To this end, studies can be down rated to moderate, low, and very low quality. On the other hand, observational studies are deemed *per se* to have low quality of evidence. Starting at the lowest rating of the two pairwise estimates (that contribute as first-order loops to the indirect estimate), the rating of indirect estimates can be further down rated for imprecision or intransitivity (dissimilarity between studies in terms of clinical or methodological characteristics) [32].

## 3. Results

### 3.1. Characteristics of Included Studies

**Figure 1:**
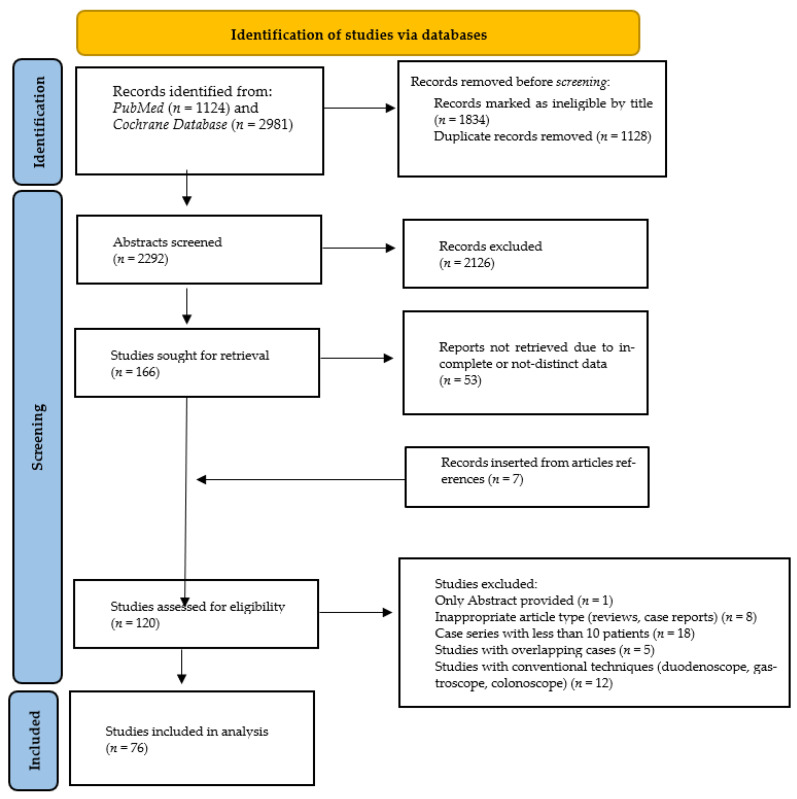
Literature research and study selection algorithm based on PRISMA guidelines.

The initial literature search resulted in 4105 studies, with 76 of them [11,13,15,17,33,34,35,36,37,38,39,40,41,42,43,44,45,46,47,48,49,50,51,52,53,54,55,56,57,58,59,60,61,62,63,64,65,66,67,68,69,70,71,72,73,74,75,76,77,78,79,80,81,82,83,84,85,86,87,88,89,90,91,92,93,94,95,96,97,98,99,100,101,102,103,104] being [11,13,15,17,32,33,34,35,36,37,38,39,40,41,42,43,44,45,46,47,48,49,50,51,52,53,54,55,56,57,58,59,60,61,62,63,64,65,66,67,68,69,70,71,72,73,74,75,76,77,78,79,80,81,82,83,84,85,86,87,88,89,90,91,92,93,94,95,96,97,98,99,100,101,102] eligible for inclusion, after assessment for exclusion criteria. Overall, 3569 ERCPs using the studied methods were included in our analysis. The flowchart for the study is shown in Figure 1 and Supplementary Table S1 summarizes the main characteristics of the included studies.

All studies, but seven [41,46,68,70,71,72,78], were retrospective, and evaluated at least one technique. Sixteen studies [15,17,92,93,94,95,96,97,98,99,100,101,102,103] provided comparisons between two or more modalities. Fifty-five studies evaluated -EA-ERCP, including 53 with BA-ERCP [11,13,15,17,33,34,35,36,37,38,39,40,41,44,45,46,47,48,49,50,51,52,53,54,56,57,58,59,60,61,62,63,64,65,66,67,68,69,70,71,72,73,92,93,95,96,97,98,101,102,103] and 4 with manual spiral enteroscope-assisted ERCP (SE-ERCP) [42,43,101,102]. The vast majority of patients (*n* = 4934, 72.9%) underwent R-en-Y anastomosis and 38.9% (*n* = 1919) of them represented bariatric cases with RYGB. Of note, studies investigating the role of EDGE [77,82,86,87,94,97,99,100] and LA-ERCP [48,74,76,78,79,80,83,84,85,88,89,90,91,94,95,99,100] exclusively enrolled RYGB cases, except for one per modality [75,81].

The male-to-female ratio was 1.2:1 and most patients had benign conditions as the indication for ERCP, with choledocholithiasis representing almost one-third of those cases. On the other hand, only 753 patients (11.1%) were clearly recorded to have malignant obstruction, thus warranting CBD decompression.

### 3.2. Quality Assessment

The quality assessment did not reveal major methodological pitfalls among the included studies. The most common shortcoming was the absence of detailed description of statistical models [34,37,38,40,44,47,48,49,50,56,58,59,60,62,66,69,71,77,80,84,85,86,88,90,91,93,104], though without impacting the overall quality. Moreover, some studies did not provide results about demographics, albeit information considering the estimation of our primary outcome was integral (Supplementary Table S3) [17,36,77,84,93,94,99,100].

### 3.3. Primary Outcome—Technical Success in Reaching the Area of Interest (Ampulla or Anastomosis)

**Figure 2:**
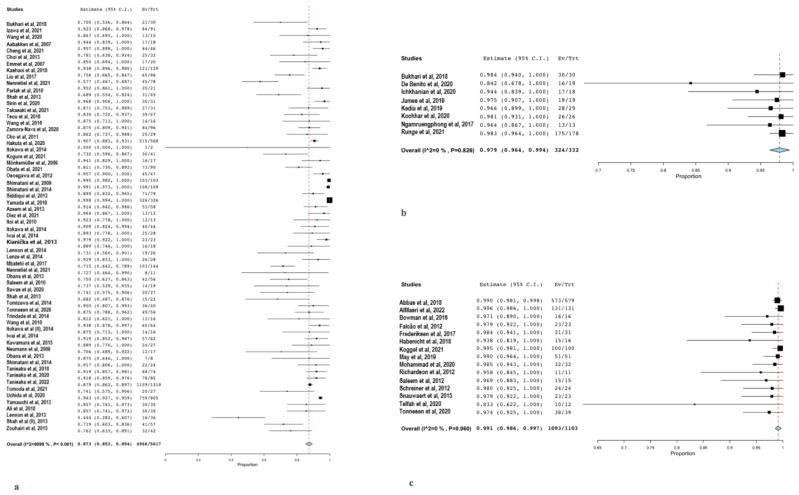
Forest plots reporting pooled results of the meta-analysis concerning technical success of (**a**) EA-ERCP, (**b**) EDGE, and (**c**) LA-ERCP.

The pooled rate of technical success for EA-ERCP (55 studies, 2549 procedures) [11,13,15,17,33,34,35,36,37,38,39,40,41,42,43,44,45,46,47,48,49,50,51,52,53,54,56,57,58,59,60,61,62,63,64,65,66,67,68,69,70,71,72,73,92,93,95,96,97,98,101,102,103] was 87.3% [95%CI: 85.3–89.4] (Figure 2a). When focusing on the individual performance of different EA-ERCP modalities (Supplementary Figure S1), SE-ERCP (4 studies, 170 procedures) [42,43,101,102] yielded a lower rate of technical success [70.3% (95%CI: 55.1–85.6)]. Moreover, single-balloon enteroscope-assisted ERCP (SBE) [15,33,34,35,36,37,38,40,45,48,50,53,54,59,62,63,64,66,68,71,72,92,93,95,101,102,103] (27 studies, 1543 procedures) provided optimal positioning in front of the ampulla and/or anastomosis in 88.1% (95%CI: 85.5–90.6) of cases and double-balloon enteroscope-assisted-ERCP (DBE) in 89.8% (95%CI: 87.1–92.4). Regarding EDGE (9 studies, 253 procedures) [77,81,82,86,87,94,97,99,100], the pooled rate of technical success was 97.9% (95%CI: 96.4–99.4); Figure 2b), while sufficient positioning in front of the ampulla was possible in 99.1% (95%CI: 98.6–99.7); Figure 2c) of the patients undergoing LA-ERCP (18 studies, 767 procedures) [48,74,75,76,78,79,80,83,84,85,88,89,90,91,94,95,99,100].

Both EDGE and LA-ERCP were shown to be superior to EA-ERCP in terms of technical success (*p* ≤ 0.001), while there was no significant difference between EDGE and LA-ERCP regarding sufficient positioning in front of the ampulla (*p* = 0.43). Finally, among the different modalities of EA-ERCP, the technical success was significantly lower among patients undergoing first-generation manual SE-ERCP when compared to DBE or SBE (*p* < 0.001), whereas BE-ERCP technical success was irrespective of the type of BE (DBE or SBE) (*p* = 0.65). Table 1 summarizes the pooled rates of technical success as well as the comparisons between the different modalities, while Supplementary Table S4 illustrates the respective comparisons among EA-ERCP modalities.

### 3.4. Secondary Outcomes

Studies evaluating EA-ERCP recorded successful duct cannulation the in 74.7% (95%CI: 71.3–78.0) of procedures, ranging between 58.8% (95%CI: 37.9–79.7) for SE-ERCP and 77.5% (95%CI: 72.4–82.6) for DBE. On the other hand, EDGE and LA-ERCP were successful in duct cannulation in 98% (95%CI: 96.5–99.6) and 98.6% (95%CI: 97.9–99.2) of cases, respectively (Supplementary Figure S2).

Regarding the therapeutic efficacy of EA-ERCP, 69.1% (95%CI: 65.3–72.9) of procedures were accompanied by a positive result, with SBE and DBE presenting similar therapeutic success rates [69.1% (95%CI: 63.8–75.5) and 71.2% (95%CI: 64.9–77.6) respectively]. The respective rate using manual SE-ERCP was 56.1% (95%CI: 32.0–80.2). The accomplishment of therapeutic outcome was also, optimally achieved using EDGE [97.9% (95%CI: 96.3–99.4), I^2^: 0%, *p =* 0.825] and LA-ERCP [98.5% (95%CI: 97.8–99.2), I^2^: 0%, *p =* 0.647; Supplementary Figure S3).

The comparisons among the different modalities (Table 1) demonstrated that the rate of duct cannulation and therapeutic efficacy were significantly lower for EA-ERCP compared to EDGE and LA-ERCP (*p* ≤ 0.001). Moreover, DBE and SBE provided equivalent results, with both being superior to manual SE-ERCP (*p* < 0.001). Finally, no difference was detected between EDGE and LA-ERCP when evaluated for access to the duct and successful therapeutic manipulations (*p* = 0.92 and 0.8 respectively).

### 3.5. Adverse Events

The highest pooled rate of adverse events was recorded among LA-ERCP cases [15.1% (95%CI: 9.40–20.8)], but was not statistically different (*p* = 0.75) from EDGE [13.1% (95%CI: 7.50–18.8)]. On the other hand, EA-ERCP resulted in significantly fewer adverse events [5.7% (95%CI: 4.50–6.80)] compared to both of the other techniques. The subgroup analysis of the individual modalities included as EA-ERCP, did not reveal any difference between SE-ERCP, SBE, and DBE. Regarding the most common ERCP-related adverse events, i.e., post-ERCP pancreatitis (PEP) and cholangitis, they presented similar prevalence among the modalities analyzed, without statistical significance at any comparison. PEP percentage ranged between 1.7% and 4.1% and cholangitis was diagnosed in 0.2–1.6% of all cases.

### 3.6. Quality of Evidence

Given that all of the included studies were observational, the quality of evidence was rated as low. No reasons for further downgrading were recognized. Therefore, based on the meta-analysis, the low quality of evidence supported the comparisons among the presented modalities.

### 3.7. Publication Bias

The visual assessment of the funnel plot to investigate any publication bias revealed relative symmetry regarding primary outcome, thus implying low possibility of publication bias for technical success (Supplementary Figure S4).

## 4. Discussion

Performing ERCP in patients with SAA can be a cumbersome task. In this study, we evaluated all available advanced ERCP techniques aiming to provide successful outcomes regardless of type of altered anatomy, in contrast to previous systematic reviews which assessed the respective modalities in various subpopulations. In this regard, the pooled results from 76 studies demonstrated that, among patients with SAA, EDGE and LA-ERCP were equivalent for all evaluated outcomes, whereas EA-ERCP was inferior to both with regards to obtaining access to the point of interest, cannulation, and provision of therapeutic benefit.

In a related study, Ayoub et al. [26] evaluated the role of LA-ERCP and EA-ERCP for the subgroup of patients with RYGB, thus indicating similar results even in this specific SAA subpopulation. Although no direct comparison between pooled rates was reported, the detection of the ampulla was successful in 98.5% (95%CI: 97.6–99.2) of cases that underwent LA-ERCP accompanied by provision of therapeutic benefit in 97.9% (95%CI: 96.7–98.7), in contrast to the lower rates with EA-ERCP [80.0% (95%CI: 71.3–87.4) and 73.2% (95%CI: 62.5–82.6) respectively] [26] Our results on LA-ERCP were similar, thus reflecting the specific pool of available studies with RYGB, whereas the respective rates of EA-ERCP revealed some fluctuations [technical success: 87.3% (95%CI: 85.3–89.4) and therapeutic success 69.1% (95%CI: 65.3–72.9) in our study], probably due to the broader spectrum of included reports. Considering adverse events, PEP and cholangitis had comparable incidence following all techniques in our study, whereas the overall adverse events were more common after interventional procedures compared to enteroscopy, thus implying an increased risk irrelevant to ERCP but mostly related to the access (LA or EUS guided). This can be attributed to the higher risk of intrabdominal infections and abscesses, perforation and hematomas that LA-ERCP carries, whereas the main pitfalls during EDGE are stent dislodgement and perforation [26].

The vast majority of included studies evaluated EA-ERCP (SE, DBE and SBE) and the endoscopic technique to approach the ampulla does not differ from conventional enteroscopy. However, manual SE-ERCP seems to provide a low rate of access to the ampulla, attributed with low cannulation and therapeutic success compared to other enteroscope-assisted modalities. This is also supported by previous data on patients who underwent RYGB surgery, especially when compared with LA-ERCP [26]. More specifically, previous generation manual SE-ERCP yielded optimal positioning in front of the ampulla in 78.9% (95%CI: 65.8–89.5), successful CBD access in 89.4% (95%CI: 51.3–98.8), and therapeutic success in 85.5 % (95% CI: 34.1–97.3 %) of cases [26]. On the other hand, in our study, balloon-assisted ERCP (DBE or SBE), provided significantly superior results, approaching 74.7% and 77.5%, respectively, in terms of canulation rates, although cannulation rate, though they still could not cover the gap with the other, invasive, techniques. In this regard, Izawa et al. [55] indicated that R-en-Y reconstruction was the main risk factor for BA-ERCP failure, thus limiting its applicability among those patients. Interestingly, Wu et al. [38] in a retrospective case study, supported that SBE yielded high success rates (93.8%) when a surgeon planned the route of the scope and monitored fluoroscopy to determine and guide the scope’s progress to the R-en-Y anastomosis. Respective modifications of the procedure are common among the included studies, whilst their retrospective design could not create a similar background for comparison, thus probably predisposing to the detected heterogeneity. Moreover, some reports indicated beneficial results using short-type SBE or DBE compared to conventional ones, but their superiority does not reflect access to the ampulla, but the duration of the ERCP and the feasibility of therapeutic manipulations using a wider range of devices [22,39,105]. Emerging data about the role of the new generation motorized spiral enteroscope in patients with altered anatomy imply a promising role for this technique in ERCP, given its safety and short learning curve [106]. Initial reports in patients warranting pancreato-biliary intervention indicated a rate of 8/10 in approaching the ampulla or the anastomosis, and in 87.5% of them cannulation was successful [107,108].

The most effective techniques, LA-ERCP and EDGE, were mainly assessed in patients having undergone RYGB, thereby preserving a detached gastric stunt. The remnant stomach is used as the substrate to gain access to the normal route via transcutaneous stoma or lumen apposing metal stents (LAMS). This approach could be modified to achieve applicability to any RY surgery. Thus, EUS-guided entero-enteral anastomosis to perform ERCP (EDEE) yielded optimal technical results in obtaining access through the LAMS (100%) and completing ERCP (94.4%). In our study, no significant difference was recorded between EDGE and LA-ERCP regarding for any outcome (*p* ≥ 0.43). Therefore, the selection should be based on further parameters, particularly local availability and expertise. LA-ERCP requires a multi-disciplinary team including surgical support and facilities. Moreover, the surgical approach could be burdened by adhesions following the previous intervention and the general condition of the patient, whereas the direct access to the abdominal cavity comprises an important advantage in case of complications. Regarding EDGE, an advanced level of EUS handling is a prerequisite to managing the complex manipulations, though it is less invasive than LA-ERCP. The main drawback is the necessity of a two-session approach to allow the maturation and stabilization of the anastomosis [99,109]. Nevertheless, the technical obstacles during EDGE could be resolved with the application of larger LAMS to inhibit dislodgement and achieve single session interventions [110] For example, the risk of stent migration using 15 mm LAMS was significantly greater compared to 20 mm stents (odds ratio: 5.36; 95%CI: 1.29–22.24; *p* ≤ 0.021). Moreover, stent fixation could, also, be a potential alternative, although further evaluation of this hypothesis is necessary [110] The background of this rationale is based on the mechanical stabilization of the LAMS, as suggested for esophageal stents [111].

Another point of consideration is the balance between efficacy and expenditures. In their study, James et al. [112], compared EA-ERCP, EDGE, and LA-ERCP in terms of cost-effectiveness among RYGB patients in the USA, based on already published retrospective studies, instead of native cases. EDGE was indicated as the most cost-effective approach, costing around half and one seventh of the per QALY compared to EA-ERCP and LA-ERCP, respectively. Furthermore, Wang et al. [94] retrospectively assessed a novel cohort to evaluate procedural and hospitalization costs of the aforementioned procedures, thus strengthening the advantage of EDGE over LA-ERCP and supporting equal results in comparison with EA-ERCP. More specifically, all patients who underwent EDGE were successfully managed and relative results whereas similar percentage was recorded for LA-ERCP (98%). However, the increased rate of adverse events and the prolonged duration of hospitalization, in addition to the procedural costs for LA-ERCP, resulted in significantly higher costs for LA-ERCP compared to EDGE (mean difference of $9700) and EA-ERCP (mean difference of $7900).

The main limitation of this review is the inclusion of retrospective non-comparative studies, thus impacting heterogeneity, especially considering EA-ERCP. The provided indirect comparison between modalities, also, downgrades the power of our results and creates some rational considerations about their clinical application. This is, also, reflected to our GRADE assessment, where the summary of evidence is classified having low quality, due to the available studies in the literature. Moreover, the investigated outcomes of applied techniques were not clearly available per surgery type in all studies, to be inserted in a respective statistical model and guide the selection of ERCP technique with regards to the history of surgery. However, the superiority of EDGE and LA-ERCP, at least for RYGB patients, could be extended to any other surgery type, given the applicability of these techniques in creating entero-enteral anastomoses. Additional choices to treat those patients include surgery, anterograde EUS drainage, percutaneous transhepatic biliary access which could be combined with cholangioscopy or rendezvous and hepatico-/pancreatico-gastrostomy. Those techniques were not included in our analysis, albeit having promising success, as they are selected for specific indications and cannot provide the entire spectrum of therapeutic results. Nevertheless, future studies with biliary drainage as the main outcome could provide a holistic assessment of all those modalities. Finally, another consideration is the variation in expertise among endoscopists, which could affect the success rates of every technique, and could not be quantified with the exception of randomized controlled studies.

To conclude, transluminal or transcutaneous access to the afferent loop of surgically modified anatomy, via EUS or laparoscopy, respectively, represents the most effective technique to perform ERCP, whereas BA- and SE-ERCP are significantly suboptimal for the respective outcomes. However, the higher prevalence of adverse events, especially with LA-ERCP, attributed to the provision of access should be considered when designing the intervention. In addition to the type of surgery, regional availability, expertise, and costs should also be factors that could guide the decision between these two modalities. Future trials should be based on similar design and distinct procedural steps to provide reliable comparisons and limited heterogeneity. Moreover, an assessment based on the type of surgery would be useful to illuminate potential differences in necessary modalities, devices and manipulations.

## Figures and Tables

**Table 1:** Pooled outcomes and comparisons between modalities.

		Comparison (*Sig.*)	
		EDGE	LA-ERCP
	Technical success rate (95%CI)		
EA-ERCP	87.3 (85.3–89.4)	0.001 *	<0.001 *
EDGE	97.9 (96.4–99.4)		*0.43*
LA-ERCP	99.1 (98.6–99.7)		
	Cannulation success rate (95%CI)		
EA-ERCP	74.7 (71.3–78.0)	<0.001 *	<0.001 *
EDGE	98 (96.5–99.6)		*0.92*
LA-ERCP	98.6 (97.9–99.2)		
	Therapeutic success rate (95%CI)		
EA-ERCP	69.1 (65.3–72.9)	<0.001 *	<0.001 *
EDGE	97.9 (96.3–99.4)		*0.80*
LA-ERCP	98.5 (97.8–99.2)		
	Adverse Events rate (95%CI)		
EA-ERCP	5.7 (4.50–6.80)	0.04 *	0.003 *
EDGE	13.1 (7.50–18.8)		*0.75*
LA-ERCP	15.1 (9.40–20.8)		

* *p* < 0.05 indicates statistically significant difference. CI, confidence interval; EA-ERCP, enteroscope-assisted ERCP; EDGE, EUS-directed transgastric ERCP; LA-ERCP, laparoscopy-assisted ERCP.

## Supplementary Materials

The following supporting information can be downloaded at: https://www.mdpi.com/article/10.3390/life12101646/s1, Table S1: Main characteristics of included studies; Table S2: PRISMA 2020 checklist of the presented objects in this review; Table S3: Quality assessment of studies; Table S4: Pooled outcomes and comparisons between enteroscope-assisted modalities; Figure S1: Forest plots reporting pooled results of the meta-analysis concerning technical success of (a) DBE, (b) SBE, and (c) manual SE-ERCP; Figure S2: Forest plots reporting pooled results of the meta-analysis concerning cannulation success of (a) EA-ERCP, (b) EDGE, and (c) LA-ERCP; Figure S3: Forest plots reporting pooled results of the meta-analysis concerning therapeutic success of (a) EA-ERCP, (b) EDGE, and (c) LA-ERCP; Figure S4: Funnel plot presenting the probability of publication bias regarding technical success.

## Abbreviations

BA-ERCP	balloon-assisted ERCP
CBD	common bile duct
DBE	double-balloon enteroscope-assisted ERCP
EA-ERCP	enteroscope-assisted ERCP
EDEE	entero-enteral anastomosis to perform ERCP
EDGE	EUS-directed transgastric ERCP
ERCP	endoscopic retrograde cholangiopancreatography
EUS	endoscopic ultrasound
LA-ERCP	laparoscopy-assisted ERCP
LAMS	lumen apposing metal stents
SE-ERCP	spiral enteroscope-assisted ERCP
RY	Roux-n-Y
RYGB	Roux-n-Y gastric bypass
SAA	surgically altered anatomy
SBE	single-balloon enteroscope-assisted ERCP
