# Supplementary material for: Comparison between Enteroscopy-, Laparoscopy- and Endoscopic Ultrasound-Assisted Endoscopic Retrograde Cholangio-Pancreatography in Patients with Surgically Altered Anatomy: A Systematic Review and Meta-Analysis
Source: Life (Basel). 2022 Oct 20;12(10):1646. doi: 10.3390/life12101646 (PMC9605390; doi:10.3390/life12101646)
Supplement: Supplementary file 1 [file life-12-01646-s001.zip › life-1932318-supplementary.pdf]

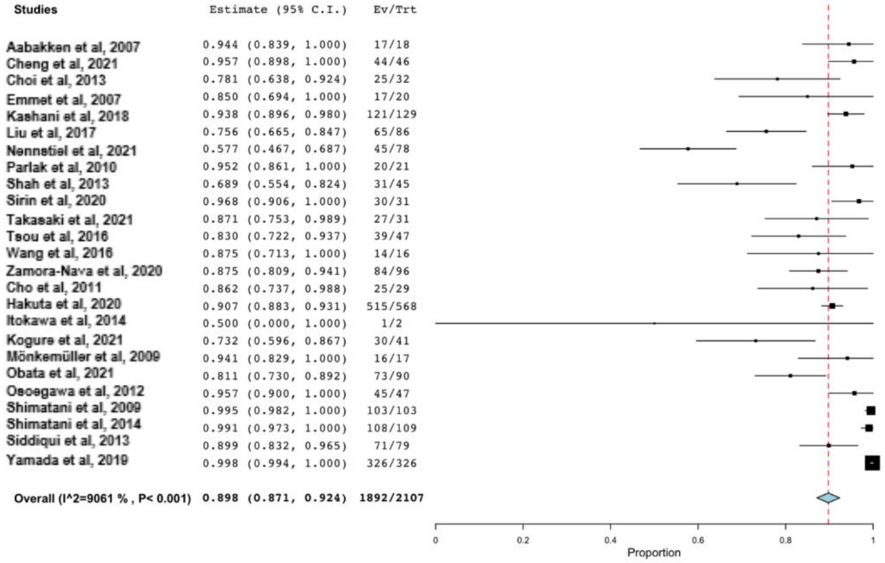

a

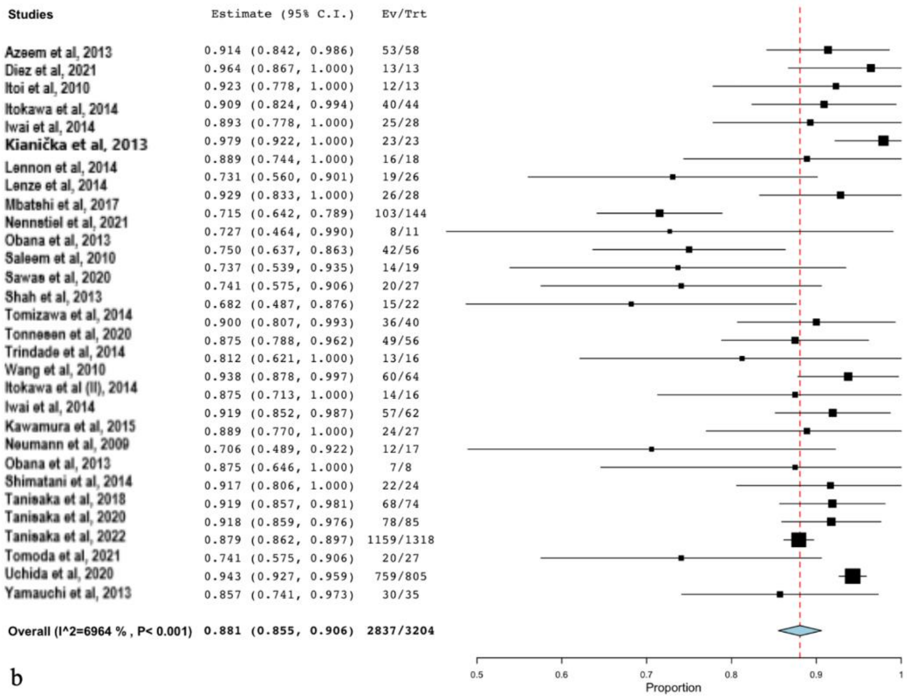

b

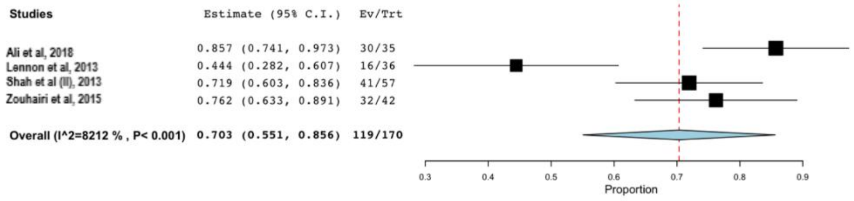

c

Figure S1. Forest plots reporting pooled results of the meta-analysis concerning technical success of (a) DBE, (b) SBE, and (c) manual SE-ERCP.

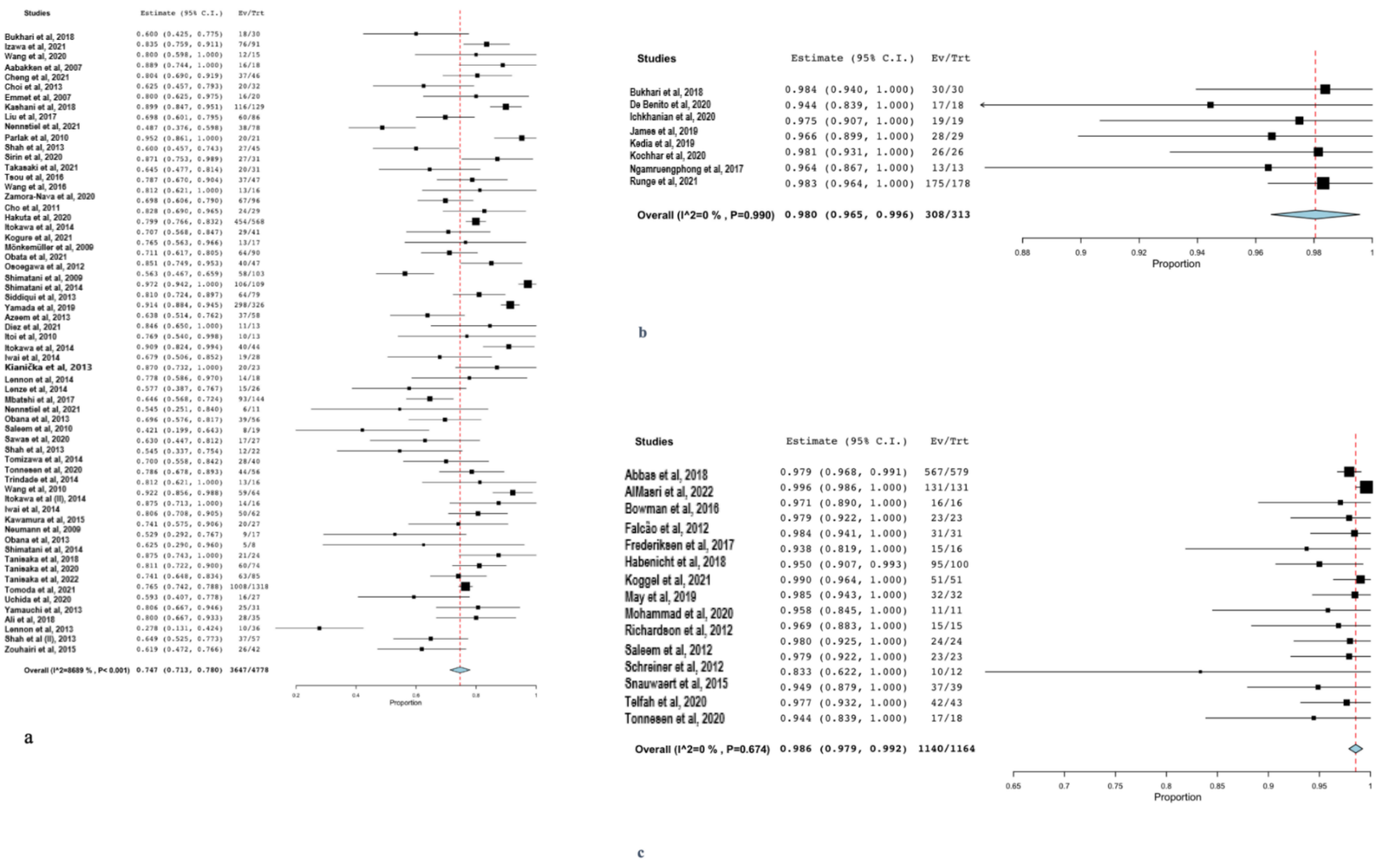

Figure S2. Forest plots reporting pooled results of the meta-analysis concerning cannulation success of (a) EA-ERCP, (b) EDGE, and (c) LA-ERCP

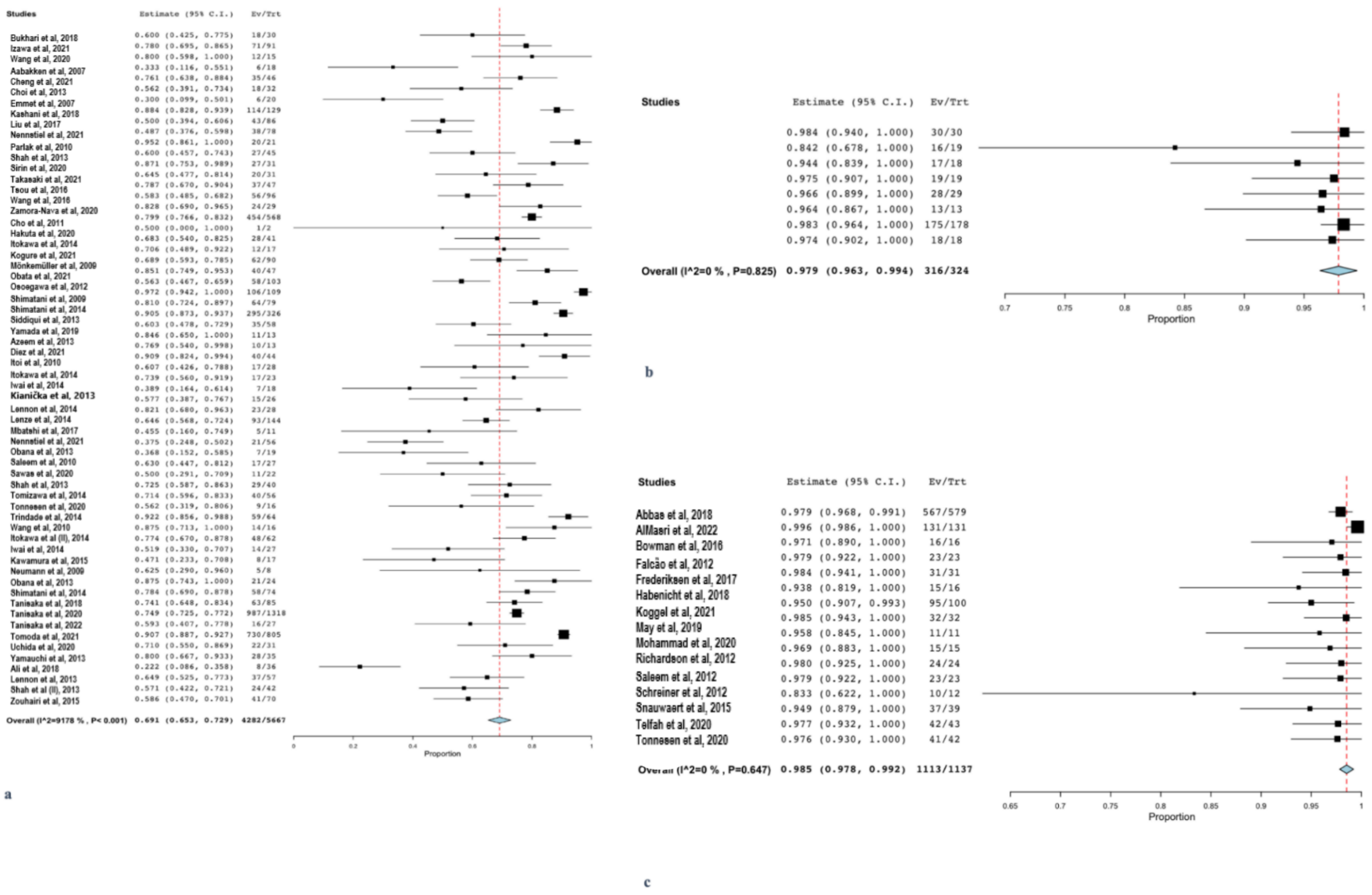

Figure S3. Forest plots reporting pooled results of the meta-analysis concerning therapeutic success of (a) EA-ERCP, (b) EDGE, and (c) LA-ERCP.

Supplementary Table S1. Main characteristics of included studies

No	Author/year	Study design	Study period	Patients, n	Endoscopic modality, n (%)	Age [mean ± SD, median (range)]	Sex, female; n(%)	Indication, n (%): lithiasis	SOD	benign stenosis	malignant stenosis	leak	abnormal LFTs/ dilatation	pancreatitis	cholangitis	other	Type of Surgery, n (%): RY	RYGB	BII	Whipple	RY-h/jstomy	other	Previous failure, n (%)	Intact papilla, n (%)
Enteroscope assisted-ERCP																								
1	Aabakken et al, 2007	case series	2005-2006	13	DBE	53(2-81)	4 (30.8)					n/a					13 (100)	0	0	0	0	0	2 (15.4)	2 (15.4)
2	Cheng et al, 2021	retrospective	2015-2020	37	DBE	70.8 (29-100)	16 (43.2)	15 (40.5)	0 (0.0)	0 (0.0)	0 (0.0)	0 (0.0)	15 (40.5)	2 (5.4)	5 (13.5)	0 (0.0)	10 (27.0)	0 (0.0)	15 (40.5)	7 (18.9)	5 (13.5)	0 (0.0)	n/a	n/a
3	Cho et al, 2011	retrospective	2007-2008	20	short DBE	58 (26-85)	10 (50.0)	10 (50.0)	0 (0.0)	8 (40.0)	0 (0.0)	1 (5.0)	0 (0.0)	0 (0.0)	8 (40.0)	2 (10.0)	5 (25.0)	0 (0.0)	6 (30.0)	1 (5.0)	7 (35.0)	1 (5.0)	12 (60.0)	n/a
4	Diez et al, 2021	case series	2018-2020	11	SBE	81 (60-91)	2 (18.2)	11 (100)	0 (0.0)	0 (0.0)	0 (0.0)	0 (0.0)	0 (0.0)	0 (0.0)	0 (0.0)	0 (0.0)	11 (100)	0 (0.0)	0 (0.0)	0 (0.0)	0 (0.0)	0 (0.0)	n/a	10 (90.9)
5	Emmet et al, 2007	case series	2005-2007	14	DBE	47 (27-73)	7 (50.0)	0 (0.0)	0 (0.0)	0 (0.0)	0 (0.0)	0 (0.0)	4 (28.6)	3 (21.4)	2 (14.3)	11 (78.6)	0 (0.0)	14 (100)	0 (0.0)	0 (0.0)	0 (0.0)	0 (0.0)	n/a	6 (42.9)
6	Hakuta et al, 2020	retrospective	2010-2019	568	short DBE	n/a	208 (36.6)	135 (23.8)	0 (0.0)	159 (28.0)	89 (15.7)	0 (0.0)	0 (0.0)	0 (0.0)	44 (7.7)	40 (7.0)	361 (63.6)	0 (0.0)	132 (23.2)	0 (0.0)	0 (0.0)	75 (13.2)	n/a	n/a
7	Itoi et al, 2010	retrospective	2007-2008	13	SBE	67.5 (36-86)	3 (23.1)	11 (84.6)	0 (0.0)	0 (0.0)	0 (0.0)	0 (0.0)	1 (7.7)	0 (0.0)	1 (7.7)	0 (0.0)	9 (69.2)	0 (0.0)	2 (15.4)	0 (0.0)	2 (15.4)	0 (0.0)	n/a	2 (15.4)
8	Itokawa et al, 2014	not stated	2005-2013	62	BA-ERCP	12-81	29 (46.8)	25 (40.3)	0 (0.0)	0 (0.0)	0 (0.0)	0 (0.0)	0 (0.0)	0 (0.0)	19 (30.6)	36 (58.1)	0 (0.0)	0 (0.0)	0 (0.0)	28 (45.2)	34 (54.8)	0 (0.0)	2 (3.2)	n/a
9	Iwai et al, 2014	retrospective	2007-2013	90	SBE<br>short SBE	n/a	n/a	28 (31.1)<br>62 (68.9)	0 (0.0)	0 (0.0)	0 (0.0)	0 (0.0)	0 (0.0)	0 (0.0)	0 (0.0)	0 (0.0)	19 (21.1)<br>52 (57.8)	0 (0.0)<br>0 (0.0)	9 (10.0)<br>10 (11.1)	0 (0.0)<br>0 (0.0)	0 (0.0)<br>0 (0.0)	0 (0.0)<br>0 (0.0)	n/a	22 (24.4)<br>41 (45.6)
10	Izawa et al, 2021	retrospective	2012-2019	91	BA-ERCP	72.6 (± 72.6)	22 (24.2)	58 (63.7)	0 (0.0)	27 (29.7)	0 (0.0)	0 (0.0)	0 (0.0)	0 (0.0)	0 (0.0)	7 (11.5)	58 (63.7)	0 (0.0)	8 (63.7)	18 (19.8)	0 (0.0)	7 (7.7)	n/a	n/a
11	Kashani et al, 2018	retrospective	2005-2012	103	DBE	50 (22-82)	90 (87.4)	26 (25.2)	66 (64.1)	8 (7.8)	0 (0.0)	8 (7.8)	5 (4.9)	9 (8.7)	0 (0.0)	1 (0.9)	0 (0.0)	103 (100)	0 (0.0)	0 (0.0)	0 (0.0)	0 (0.0)	n/a	92 (89.3)
12	Kawamura et al, 2015	retrospective	2012-2013	18	short SBE	17 (47-86)	4 (22.2)	6 (33.3)	0 (0.0)	3 (16.7)	17 (94.4)	0 (0.0)	0 (0.0)	0 (0.0)	0 (0.0)	1 (5.6)	15 (83.3)	0 (0.0)	2 (11.1)	0 (0.0)	10 (6.1)	0 (0.0)	n/a	7 (38.9)
13	Kianička et al, 2013	retrospective	2009-2011	15	SBE	53.8 (26-67)	8 (53.3)	5 (33.3)	0 (0.0)	5 (33.3)	0 (0.0)	0 (0.0)	1 (1.7)	0 (0.0)	0 (0.0)	0 (0.0)	0 (0.0)	0 (0.0)	0 (0.0)	0 (0.0)	15 (100)	0 (0.0)	n/a	n/a
14	Kogure et al, 2021	retrospective	2006-2019	38	short DBE	66 (47-72)	18 (47.4)	ERP: 38 (100)									13 (34.2)		25 (65.8)				3 (7.9)	n/a
15	Lenze et al, 2014	prospective	2008-2011	26	SBE	69.5 (20–86)	10 (38.5)	10 (38.5)	0 (0.0)	9 (34.6)	7 (26.9)	0 (0.0)	0 (0.0)	0 (0.0)	0 (0.0)	0 (0.0)	23 (88.5)			3 (11.5)			26 (100)	14 (53.8)
16	Liu et al, 2017	retrospective	2009-2015	51	DBE	60.5 (18-89)	12 (23.5)	0 (0.0)	0 (0.0)	0 (0.0)	0 (0.0)	3 (3.5) *	63 (73.3)	1 (1.2)	14 (16.3)	19 (22.1)	51 (100)	0 (0.0)	0 (0.0)	0 (0.0)	0 (0.0)	0 (0.0)	n/a	9 (17.6)
17	Mönkemüller et al, 2009	prospective	n/a	11	sDBE	59.7 (36-77)	1 (9.1)	1 (9.1)	0 (0.0)	1 (9.1)	2 (18.2)	0 (0.0)	4 (36.4)	0 (0.0)	2 (18.2)	1 (9.1)	11 (100)	0 (0.0)	0 (0.0)	0 (0.0)	0 (0.0)	0 (0.0)	n/a	2 (18.2)
18	Neumann et al, 2009	prospective	2007-2008	13	short SBE	66.5 (25-77)	5 (38.5)	4 (30.8)	0 (0.0)	0 (0.0)	0 (0.0)	0 (0.0)	3 (23.1)	0 (0.0)	6 (46.2)	0 (0.0)	13 (100)	0 (0.0)	0 (0.0)	0 (0.0)	0 (0.0)	0 (0.0)	n/a	4 (30.8)
19	Obana et al, 2013	n/a but probably prospective	2008-2012	19	SBE<br>short SBE	75.1 ± 8.4<br>78.5 ± 4.9	3 (15.8)<br>1 (5.3)	15 (78.9)	0 (0.0)	0 (0.0)	4 (21.1)	0 (0.0)	0 (0.0)	0 (0.0)	0 (0.0)	0 (0.0)	19 (100)	0 (0.0)	0 (0.0)	0 (0.0)	0 (0.0)	0 (0.0)	n/a	n/a
20	Obata et al, 2021	retrospective	2011-2020	79	short DBE	79 (73-84)	17 (21.1)	79 (100)	0 (0.0)	0 (0.0)	0 (0.0)	0 (0.0)	0 (0.0)	0 (0.0)	0 (0.0)	0 (0.0)	79 (100)	0 (0.0)	0 (0.0)	0 (0.0)	0 (0.0)	0 (0.0)	n/a	n/a
21	Osoegawa et al, 2012	prospective	2006-2011	28	short DBE	74 (54-91)	10 (35.7)	n/a									13 (46.4)	0 (0.0)	15 (53.6)	0 (0.0)	0 (0.0)	0 (0.0)	n/a	26 (92.9)
22	Parlak et al, 2010	retrospective	n/a	14	DBE	45.3 (28-61)	7 (50.0)	0 (0.0)	0 (0.0)	1 (7.1)	0 (0.0)	0 (0.0)	3 (21.4)	0 (0.0)	9 (64.3)	1 (7.1)	14 (100)	0 (0.0)	0 (0.0)	0 (0.0)	0 (0.0)	0 (0.0)	n/a	n/a
23	Saleem et al, 2010	retrospective	2008-2010	50	SBE	57 (19–85)	16 (32.0)					n/a					41 (73.2) *	15 (26.8)*	0 (0.0)	0 (0.0)	0 (0.0)	0 (0.0)	0 (0.0)	0 (0.0)
24	Shimatani et al, 2009	retrospective	2006-2008	68	short DBE	n/a	n/a					n/a					36 (52.9)	0 (0.0)	17 (25.0)	15 (22.1)	0 (0.0)	0 (0.0)	n/a	55 (80.9)
25	Shimatani et al, 2014	retrospective	2016	24	short SBE	n/a	n/a					n/a					12 (50.0)	0 (0.0)	3 (12.5)	9 (37.5)	0 (0.0)	0 (0.0)	n/a	7 (29.2)

26	Siddiqui et al, 2013	retrospective	2008-2011	79	short DBE	58 (29-86)	49 (62.0)	48 (60.8)	3 (3.8)	20 (25.3)	0 (0.0)	3 (3.8)	0 (0.0)	0 (0.0)	0 (0.0)	5 (6.3)	12 (15.2)	39 (49.4)	3 (3.8)	20 (25.3)	5 (6.3)	0 (0.0)	8 (10.1)	42 (53.2)
27	Takasaki et al, 2021	retrospective	2010-2020	31	DBE	67.4 ± 16.8	7 (22.6)	31 (100)	0 (0.0)	0 (0.0)	0 (0.0)	0 (0.0)	0 (0.0)	0 (0.0)	0 (0.0)	0 (0.0)	16 (51.6)	0 (0.0)	1 (3.2)	5 (16.1)	9 (29.0)	0 (0.0)	n/a	n/a
28	Tanisaka et al, 2018	retrospective	2011-2017	61	short SBE	71 (64.5–76.5)	15 (24.6)	41 (67.2)	0 (0.0)	0 (0.0)	20 (32.8)	0 (0.0)	0 (0.0)	0 (0.0)	0 (0.0)	0 (0.0)	61 (100)	0 (0.0)	0 (0.0)	0 (0.0)	0 (0.0)	0 (0.0)	n/a	58 (95.1)
29	Tanisaka et al, 2020	retrospective	2011-2018	85	short SBE	71.5 (36-88)*	17 (21.8)**	53 (67.9)**	0 (0.0)	0 (0.0)	25 (32.1)	0 (0.0)	n/a	0 (0.0)	0 (0.0)	85 (100)	0 (0.0)	0 (0.0)	0 (0.0)	0 (0.0)	0 (0.0)	0 (0.0)	n/a	85 (100)
30	Tanisaka et al, 2022	retrospective	2011-2019	1318	short SBE	73 (66-79)	398 (30.2)	Benign indication: 1069 (81.1), Malignant indication: 249 (18.9)									527 (40.0)	0 (0.0)	200 (15.2)	356 (27.0)	221 (16.8)	14 (1.0)	n/a	674 (51.1)
31	Tomizawa et al, 2014	retrospective	2009-2011	14	SBE	63 (35-83)	9 (64.3)	0 (0.0)	0 (0.0)	0 (0.0)	0 (0.0)	0 (0.0)	10 (71.4)	0 (0.0)	7 (50.0)	5 (35.7)	14 (100)	0 (0.0)	0 (0.0)	0 (0.0)	0 (0.0)	0 (0.0)	0 (0.0)	2 (14.3)
32	Tomoda et al, 2021	retrospective	2011-2019	45	short DBE	67 (59-75)	16 (35.6)	0 (0.0)	0 (0.0)	0 (0.0)	27 (60.0)	0 (0.0)	0 (0.0)	0 (0.0)	0 (0.0)	0 (0.0)	22 (48.9)	0 (0.0)	5 (11.1)	18 (40.0)	0 (0.0)	0 (0.0)	n/a	18 (40.0)
33	Trindade et al. 2014	retrospective	2011-2013	56	SBE (+cap)	56 (28-80)	45 (80.4)	29 (51.8)	0 (0.0)	4 (7.1)	0 (0.0)	4 (7.1)	9 (16.1)	0 (0.0)	10 (17.9)	0 (0.0)	n/a	n/a	n/a	n/a	n/a	n/a	n/a	44 (78.6)
34	Tsou et al, 2016	retrospective	2007-2013	47	DBE	54.4 (26–87)	25 (53.2)	35 (74.5)	0 (0.0)	6 (12.8)	6 (12.8)	0 (0.0)	0 (0.0)	0 (0.0)	0 (0.0)	0 (0.0)	14 (29.8)	0 (0.0)	0 (0.0)	0 (0.0)	33 (70.2)	0 (0.0)	n/a	13 (27.7)
35	Uchida et al, 2020	retrospective	2011-2018	319	short DBE	69 (median)	124 (38.9)	58 (18.2)	0 (0.0)	221 (69.3)	0 (0.0)	0 (0.0)	0 (0.0)	0 (0.0)	0 (0.0)	30 (9.4)	135 (42.3)	0 (0.0)	22 (6.9)	162 (50.8)	0 (0.0)	0 (0.0)	n/a	41 (12.9)
36	Wang et al, 2010	retrospective	2007-2009	13	SBE	54 (28-82)	11 (84.6)	1 (7.7)	0 (0.0)	2 (15.4)		0 (0.0)	7 (53.8)	2 (15.4)	4 (30.8)	0 (0.0)	3 (18.8) *	8 (50.0)*	1 (6.2)*	3 (18.8)*	1 (6.2)*	0 (0.0)	n/a	n/a
37	Wang et al, 2020	retrospective	2016-2019	15	BA-ERCP	51.5 (28-63)	4 (26.7)	0 (0.0)	0 (0.0)	13 (86.7)	1 (6.7)	0 (0.0)	0 (0.0)	0 (0.0)	0 (0.0)	0 (0.0)	1 (6.7)	0 (0.0)	0 (0.0)	4 (26.7)	9 (60.0)	0 (0.0)	n/a	n/a
38	Wu et al, 2019	retrospective	2010-2016	46	SBE	72.1 ± 11.2	19 (41.3)	32 (69.6)	0 (0.0)	20 (43.5)	6 (13.0)	0 (0.0)	0 (0.0)	0 (0.0)	0 (0.0)	10 (21.7)	46 (100)	0 (0.0)	0 (0.0)	0 (0.0)	0 (0.0)	0 (0.0)	n/a	n/a
39	Yamada et al, 2019	retrospective	2005-2017	322	short DBE	65.15 ± 15.6	142 (44.1)	149 (46.3)	185 (57.5)	0 (0.0)	0 (0.0)	0 (0.0)	0 (0.0)	0 (0.0)	0 (0.0)	11 (3.4)	82 (25.5)	0 (0.0)	82 (25.5)	0 (0.0)	158 (49.1)	0 (0.0)	n/a	n/a
40	Yamauchi et al, 2013	retrospective	2011-2012	28	short SBE	72.0 (50-85)	2 (7.1)	19 (67.9)	0 (0.0)	0 (0.0)	9 (32.1)	0 (0.0)	0 (0.0)	1 (3.6)	2 (7.1)	1 (3.6)	14 (50.0)	22 (78.6)	0 (0.0)	0 (0.0)	2 (7.1)	0 (0.0)	n/a	19 (67.9)
41	Zamora-Nava et al, 2020	prospective	2010-2016	75	DBE (mixed)	40.8 (± 10.2)	69 (71.9)*	0 (0.0)	0 (0.0)	0 (0.0)	0 (0.0)	0 (0.0)	0 (0.0)	0 (0.0)	47 (48.9)*	49 (51.0)*	81 (84.3) *	2 (2.1)*	3 (3.1)*	10 (10.4)*	0 (0.0)	0 (0.0)	26 (37.7)*	n/a
42	Ali et al, 2018	retrospective	2009-1016	31	Manual SE-ERCP	55 (22-75)	25 (80.6)	14 (45.2)	5 (16.1)	2 (6.5)	6 (19.4)	1 (3.2)	0 (0.0)	2 (6.5)	0 (0.0)	5 (16.1)	31 (100)	0 (0.0)	0 (0.0)	0 (0.0)	0 (0.0)	0 (0.0)	n/a	24 (77.4)
43	Zouhairi et al, 2015	retrospective	2009-2012	36	Manual SE-ERCP	49.3 (29-75)	34 (94.4)	13 (36.1)	4 (11.1)	20 (55.6)	0 (0.0)	2 (5.6)	1 (2.8)	0 (0.0)	0 (0.0)	0 (0.0)	33 (91.7)	0 (0.0)	2 (5.6)	0 (0.0)	1 (2.8)	0 (0.0)	n/a	n/a
Studies with EDGE																								
44	De Benito et al, 2020	retrospective	2016-2019	14	EDGE	56 (± 9.7)	12 (85.7)	6 (42.9)	0 (0.0)	0 (0.0)	0 (0.0)	1 (7.1)	2 (14.2)	2 (14.2)	3 (21.4)	0 (0.0)	0 (0.0)	14 (100)	0 (0.0)	0 (0.0)	0 (0.0)	0 (0.0)	n/a	n/a
45	Ichkhanian et al, 2020	retrospective	2014-2018	18	EDEE	63.1 (43-83)	13 (72.2)	5 (27.8)	0 (0.0)	4 (22.2)	8 (44.4)	0 (0.0)	0 (0.0)	0 (0.0)	0 (0.0)	1 (5.6)	6 (33.3)	0 (0.0)	0 (0.0)	10 (55.6)	0 (0.0)	2 (11.1)	16 (88.9)	n/a
46	James et al, 2019	retrospective	2016-2018	19	EDGE	55.5 (± 3.2)	15 (78.9)	8 (42.1)	0 (0.0)	4 (21.1)	0 (0.0)	0 (0.0)	1 (5.3)	6 (31.6)	0 (0.0)	0 (0.0)	0 (0.0)	15 (100)	0 (0.0)	0 (0.0)	0 (0.0)	0 (0.0)	3 (15.8)	n/a
47	Ngamruengphong et al, 2017	retrospective	2014-2016	13	EDGE	55 ± 16	11 (84.6)	5 (38.5)	0 (0.0)	2 (15.4)	0 (0.0)	2 (15.4)	0 (0.0)	0 (0.0)	0 (0.0)	0 (0.0)	0 (0.0)	13 (100)	0 (0.0)	0 (0.0)	0 (0.0)	0 (0.0)	n/a	n/a
48	Runge et al, 2021	retrospective	2014-2019	178	EDGE	58± 11	140 (78.7)	97 (54.5)	12 (6.7)	16 (8.9)	8 (4.5)	18 (10.1)	8 (4.5)	13 (7.3)	0 (0.0)	6 (3.4)	0 (0.0)	178 (100)	0 (0.0)	0 (0.0)	0 (0.0)	0 (0.0)	n/a	n/a
Studies with LA-ERCP																								
49	Abbas et al, 2018	retrospective	2005-2016	579	LA-ERCP	51 (43-61)	91 (15.7)	254 (43.9)	0 (0.0)	122 (21.1)	7 (1.2)	7 (1.2)	126 (21.8)	56 (9.7)	0 (0.0)	23	0 (0.0)	579 (100)	0 (0.0)	0 (0.0)	0 (0.0)	0 (0.0)	151 (26.1)	n/a
50	AlMasri et al, 2022	retrospective	2007-2019	131	LA-ERCP	60 (50-67)	106 (80.9)	102 (77.9)	23 (17.6)	0 (0.0)	3 (2.3)	3 (2.3)	0 (0.0)	0 (0.0)	0 (0.0)	0 (0.0)	131 (100)						n/a	n/a
51	Bowman et al, 2016	retrospective	n/a	15	LA-ERCP	48.8 (25-69)	11 (73.3)	1 (6.7)	0 (0.0)	4 (26.7)	0 (0.0)	0 (0.0)	0 (0.0)	5 (33.3)	0 (0.0)	5 (33.3)	0 (0.0)	15 (100)	0 (0.0)	0 (0.0)	0 (0.0)	0 (0.0)	4 (26.7)	14 (93.3)
52	Falcão et al, 2012	prospective	2003-2010	23	LA-ERCP	35.3 (27-52)	19 (82.6)	14 (60.9)	0 (0.0)	0 (0.0)	0 (0.0)	0 (0.0)	3 (13.0)	0 (0.0)	0 (0.0)	6 (26.1)	0 (0.0)	23 (100)	0 (0.0)	0 (0.0)	0 (0.0)	0 (0.0)	n/a	23 (100)
53	Frederiksen et al, 2017	retrospective	2010-2016	29	LA-ERCP	46 (25-65)	25 (86.2)	29 (100)	0 (0.0)	0 (0.0)	0 (0.0)	0 (0.0)	0 (0.0)	0 (0.0)	0 (0.0)	0 (0.0)	0 (0.0)	29 (100)	0 (0.0)	0 (0.0)	0 (0.0)	0 (0.0)	n/a	n/a
54	Habenicht et al, 2018	retrospective	2012-2016	16	LA-ERCP	55.8 (29-67)	n/a	n/a	n/a	n/a	n/a	n/a	n/a	n/a	n/a	n/a	0 (0.0)	16 (100)	0 (0.0)	0 (0.0)	0 (0.0)	0 (0.0)	3 (18.8)	n/a
55	Koggel et al, 2021	retrospective	2009-2019	86	LA-ERCP	53.5 (27–72)	70 (81.4)	61 (70.9)	2 (2.3)	0 (0.0)	0 (0.0)	7 (8.1)	0 (0.0)	0 (0.0)	25 (29.1)	5 (5.8)	0 (0.0)	86 (100)	0 (0.0)	0 (0.0)	0 (0.0)	0 (0.0)	n/a	n/a
56	May et al, 2019	retrospective	2009-2016	51	LA-ERCP	55.4 (10.9)	45 (88.2)	24 (47.1)	22 (43.1)	3 (5.9)	0 (0.0)	1 (1.9)	0 (0.0)	0 (0.0)	0 (0.0)	1 (1.9)	0 (0.0)	51 (100)	0 (0.0)	0 (0.0)	0 (0.0)	0 (0.0)	n/a	n/a
57	Mohammad et al, 2020	retrospective	2008-2016	32	LA-ERCP	54 (26-74)	26 (81.3)	16 (50.0)	0 (0.0)	0 (0.0)	0 (0.0)	1 (3.1)	11 (34.3)	2 (6.3)	2 (6.3)	0 (0.0)	0 (0.0)	32 (100)	0 (0.0)	0 (0.0)	0 (0.0)	0 (0.0)	n/a	n/a

58	Richardson et al, 2012	retrospective	2006-2011	11	LA-ERCP	54.3 (32-69)	9 (81.8)	4 (36.4)	1 (9.1)	0 (0.0)	0 (0.0)	0 (0.0)	3 (27.3)	3 (27.3)	0 (0.0)	0 (0.0)	0 (0.0)	11 (100)	0 (0.0)	0 (0.0)	0 (0.0)	0 (0.0)	4 (36.4)	n/a
59	Saleem et al, 2012	retrospective	2005-2020	15	LA-ERCP	50.9 (25-70)	12 (80.0)	3 (20.0)	9 (60.0)	1 (6.7)	0 (0.0)	1 (6.7)	0 (0.0)	1 (6.7)	0 (0.0)	0 (0.0)	0 (0.0)	15 (100)	0 (0.0)	0 (0.0)	0 (0.0)	0 (0.0)	n/a	15 (100)
60	Schreiner et al, 2012	retrospective	2007-2011	24	LA-ERCP	52	19 (79.2)	3 (12.5)	0 (0.0)	20 (83.3)	1 (4.2)	0 (0.0)	0 (0.0)	0 (0.0)	0 (0.0)	0 (0.0)	0 (0.0)	24 (100)	0 (0.0)	0 (0.0)	0 (0.0)	0 (0.0)	n/a	n/a
61	Snauwaert et al, 2015	retrospective	2008-2014	23	LA-ERCP	54 (26-79)	18 (78.3)	5 (21.7)	0 (0.0)	0 (0.0)	0 (0.0)	0 (0.0)	3 (13.0)	4 (17.4)	3 (13.0)	4 (17.4)	0 (0.0)	23 (100)	0 (0.0)	0 (0.0)	0 (0.0)	0 (0.0)	n/a	23 (100)
62	Telfah et al, 2020	retrospective	2010-2019	12	LA-ERCP	64 (34-73)	9 (75.0)	0 (0.0)	0 (0.0)	0 (0.0)	0 (0.0)	0 (0.0)	8 (66.7)	0 (0.0)	4 (33.3)	0 (0.0)	0 (0.0)	12 (100)	0 (0.0)	0 (0.0)	0 (0.0)	0 (0.0)	n/a	n/a
Studies comparing two or more techniques																								
63	Azeem et al, 2013	retrospective cohort (cross-over on modality failure)	2002-2012	36	colonoscope<br>SBE	49 (2-68)<br>54.4 (18-68)	28 (34.1)<br>11 (30.6)	0 (0.0)<br>0 (0.0)	0 (0.0)<br>0 (0.0)	64 (45.4)<br>23 (39.7)	0 (0.0)<br>0 (0.0)	3 (2.1)<br>1 (1.7)	48 (34.0)<br>22 (37.9)	0 (0.0)<br>0 (0.0)	18 (12.8)<br>10 (17.2)	8 (5.7)<br>2 (3.4)	99 (100)	0 (0.0)	0 (0.0)	0 (0.0)	0 (0.0)	0 (0.0)	n/a	n/a
64	Mbatshi et al, 2017	retrospective	2006-2015	38	classic ERCP<br>SBE<br>pediatric colonoscope	73.7 (48-91)	17 (44.7)	20 (52.6)	0 (0.0)	0 (0.0)	1 (2.6)	0 (0.0)	0 (0.0)	8 (21.1)	8 (21.1)	2 (5.3)	0 (0.0)	0 (0.0)	38 (100)	0 (0.0)	0 (0.0)	0 (0.0)	8 (21.1)	n/a
65	Nennstiel et al, 2021	retrospective	2006-2014	411	simple duodenoscope<br>pediatric colonoscope<br>SBE<br>DBE<br>colonoscope<br>gastroscope	64 (± 15)	159 (38.7)	Malignant obstruction 203 (49.4) , Benign obstruction 187 (45.5), Unclear 15 (3.6)									186 (45.3)	0 (0.0)	105 (25.5)	120 (29.2)	0 (0.0)	0 (0.0)	n/a	n/a
66	Wang et al, 2016	retrospective	2013-2016	97	gastroscope<br>colonoscope<br>simple duodenoscope<br>DBE	28-84	38 (39.2)	60 (61.9)	0 (0.0)	8 (8.2)	29 (29.9)	0 (0.0)	0 (0.0)	0 (0.0)	0 (0.0)	0 (0.0)	20 (20.6)	0 (0.0)	52 (53.6)	25 (25.8)		0 (0.0)	n/a	72 (74.2)
67	Sirin et al, 2020	retrospective	2008-2014	62	simple duodenoscope<br>DBE	61 (31-78)	21 (33.9)	23 (37.1)	0 (0.0)	6 (9.7)	9 (14.5)	0 (0.0)	9 (14.5)	0 (0.0)	0 (0.0)	0 (0.0)	11 (17.7)	0 (0.0)	51 (82.3)	0 (0.0)	0 (0.0)	0 (0.0)	31 (50.0)	n/a
68	Bukhari et al, 2018	retrospective	2014-2016	60	EDGE<br>BA-ERCP	52.5±13.4<br>61.8±11.5	27 (45.0)<br>18 (30.0)	43 (71.7)	3 (5.0)	7 (11.7)	0 (0.0)	3 (5.0)	0 (0.0)	4 (6.7)	0 (0.0)	0 (0.0)	0 (0.0)	60 (100)	0 (0.0)	0 (0.0)	0 (0.0)	0 (0.0)	n/a	n/a
69	Tonnesen et al, 2020	retrospective	2013-2017	68	LA-ERCP<br>SBE	48.8 (35-77)<br>51 (37-72)	27 (39.7)<br>26 (38.2)	48 (70.6)	0 (0.0)	4 (5.9)	0 (0.0)	7 (10.3)	0 (0.0)	0 (0.0)	0 (0.0)	20 (29.4)	0 (0.0)	68 (100)	0 (0.0)	0 (0.0)	0 (0.0)	0 (0.0)	n/a	n/a
70	Wang et al, 2021	retrospective	2016-2019	130	BA-ERCP<br>LA-ERCP<br>EDGE	55.3 (14.3)<br>50.6 (15.9)<br>59.3 (6.5)	47 (36.2)<br>38 (29.2)<br>16 (12.3)	59 (45.4)	6 (4.6)	22 (16.9)	11 (8.5)	13 (10.0)	0 (0.0)	5 (3.8)	14 (10.8)	0 (0.0)	0 (0.0)	130 (100)	0 (0.0)	0 (0.0)	0 (0.0)	0 (0.0)	n/a	n/a
71	Choi et al, 2013	retrospective comparative trial	2005-2011	72	ERCP-gastrostomy<br>DBE	44.8 ± 11.7<br>56.1 ± 12.2	42 (58.3)<br>26 (36.1)	18 (25.0)	40 (55.6)	0 (0.0)	4 (5.6)	2 (2.8)	0 (0.0)	8 (11.1)	0 (0.0)	0 (0.0)	0 (0.0)	72 (100)	0 (0.0)	0 (0.0)	0 (0.0)	0 (0.0)	n/a	n/a
72	Sawas et al, 2020	retrospective	2015-2017	30	SBE<br>RGA	62.2 (10.2)	14 (46.7)<br>11 (36.7)	16 (53.3)	0 (0.0)	1 (3.3)	1 (3.3)	1 (3.3)	9 (30.0)	3 (10.0)	0 (0.0)	0 (0.0)	0 (0.0)	30 (100)	0 (0.0)	0 (0.0)	0 (0.0)	0 (0.0)	3 (10.0)	30 (100)
73	Lennon et al, 2012	retrospective	2007-2011	54	SBE	n/a	26 (48.1)	n/a									10 (18.5)	8 (14.8)	0 (0.0)	0 (0.0)	0 (0.0)	0 (0.0)	12 (22.2)	9 (16.7)

					SE-ERCP												15 (27.8)	21 (38.9)	0 (0.0)	0 (0.0)	0 (0.0)	0 (0.0)		
74	Shah et al, 2013	retrospective	2008-2009	129	SBE<br>DBE<br>SE-ERCP	54 (20-84)	93 (72.1)	0 (0.0)	0 (0.0)	0 (0.0)	0 (0.0)	0 (0.0)	94 (72.9)	8 (6.2)	20 (15.5)	7 (5.4)	6 (4.7)	63 (48.8)	0 (0.0)	31 (24.0)	24 (18.6)	5 (3.9)	37 (28.7)	73 (56.6)
75	Kedia et al, 2019	retrospective	2005-2017	72	LA-ERCP<br>EDGE	55 (33-82)<br>56 (35-82)	36 (50.0)<br>25 (34.7)					Biliary: 49 (68.1)<br>Pancreatic: 13 (18.1)					0 (0.0)	72 (100)	0 (0.0)	0 (0.0)	0 (0.0)	0 (0.0)	9 (12.5)	72 (100)
76	Kochhar et al, 2020	retrospective-comparative	2015-2019	56	EDGE<br>LA-ERCP<br>E-ERCP	60.77±11.44<br>60.78±12.67<br>68.58±15.09	20 (35.7)<br>12 (21.4)<br>8 (14.3)	26 (51.8)	0 (0.0)	5 (8.9)	0 (0.0)	5 (8.9)	7 (12.5)	6 (10.7)	4 (7.1)	4 (7.1)	0 (0.0)	56 (100)	0 (0.0)	0 (0.0)	0 (0.0)	0 (0.0)	4 (7.1)	n/a

* calculated compared to total procedures

**calculated based on cases with technical success

BII, Billroth II; BA-ERCP, balloon assisted-ERCP; DBE, double-balloon enteroscope assisted ERCP; EA-ERCP, enteroscope assisted ERCP; EDEE; entero-enteral anastomosis to perform ERCP; EDGE, EUS-directed transgastric ERCP; ERCP, endoscopic retrograde cholangio-pancreatography; h/jstomy, hepaticojejunostomy; LA-ERCP, laparoscopy assisted- ERCP; LFTs, liver function tests; RGA, Rendezvous guidewire assisted; RY, Roux-n-Y; RYGB, Roux-n-Y gastric by-pass; SD, standard deviation; SBE, single-balloon enteroscope assisted ERCP; SE-ERCP, spiral enteroscope assisted ERCP; SOD, sphincter of Oddi disfunction

Supplementary Table S3. The results of quality assessment based on the National Heart, Lung, and Blood Institute tool for case-series

Studies	Quality assessment							
**Author, year**	**Was the study question or objective clearly stated?**	**Was the study population clearly and fully described, including a case definition?**	**Were the cases consecutive?**	**Were the subjects comparable?**	**Was the intervention clearly described?**	**Were the outcome measures clearly defined, valid, reliable, and implemented consistently across all study participants?**	**Was the length of follow-up adequate?**	**Were the statistical methods well-described?**	**Were the results well-described?**
Aabakken et al, 2007	No	Yes	Yes	Yes	Yes	Yes	Yes	No	Yes
Cheng et al, 2021	Yes	Yes	Yes	Yes	Yes	Yes	Yes	Yes	Yes
Cho et al, 2011	No	Yes	Yes	Yes	Yes	Yes	Yes	No	Yes
Diez et al, 2021	Yes	Yes	Yes	Yes	Yes	Yes	Yes	No	Yes
Emmet et al, 2007	Yes	Yes	Yes	Yes	Yes	Yes	Yes	No	Yes
Hakuta et al, 2020	Yes	Yes	Yes	Yes	Yes	Yes	Yes	Yes	Yes
Itoi et al, 2010	No	Yes	Yes	Yes	Yes	Yes	Yes	No	Yes
Itokawa et al, 2014	Yes	Yes	Yes	Yes	Yes	Yes	Yes	Yes	Yes
Iwai et al, 2014	Yes	Yes	Yes	Yes	Yes	Yes	Yes	Yes	Yes
Izawa et al, 2021	Yes	Yes	Yes	Yes	Yes	Yes	Yes	Yes	Yes
Kashani et al, 2018	Yes	Yes	Yes	Yes	Yes	Yes	Yes	Yes	Yes
Kawamura et al, 2015	No	Yes	Yes	Yes	Yes	Yes	Yes	No	Yes
Kianička et al, 2013	No	Yes	Yes	Yes	Yes	Yes	Yes	No	Yes
Kogure et al, 2021	Yes	Yes	Yes	Yes	Yes	Yes	Yes	Yes	Yes
Lenze et al, 2014	Yes	Yes	Yes	Yes	Yes	Yes	Yes	Yes	Yes
Liu et al, 2017	No	Yes	Yes	Yes	Yes	Yes	Yes	Yes	Yes
Mönkemüller et al, 2009	Yes	Yes	Yes	Yes	Yes	Yes	Yes	Yes	Yes
Neumann et al, 2009	Yes	Yes	Yes	Yes	Yes	Yes	Yes	No	Yes
Obana et al, 2013	Yes	Yes	Yes	Yes	Yes	Yes	Yes	Yes	Yes
Obata et al, 2021	Yes	Yes	Yes	Yes	Yes	Yes	Yes	Yes	Yes
Osoegawa et al, 2012	No	Yes	Yes	Yes	Yes	Yes	Yes	Yes	Yes
Parlak et al, 2010	No	Yes	Yes	Yes	Yes	Yes	Yes	No	Yes

Saleem et al, 2010	Yes	Yes	Yes	Yes	Yes	Yes	Yes	No	Yes
Shimatani et al, 2009	Yes	Yes	Yes	Yes	Yes	Yes	Yes	No	Yes
Shimatani et al, 2014	Yes	Yes	Yes	Yes	Yes	Yes	Yes	No	Yes
Siddiqui et al, 2013	Yes	Yes	Yes	Yes	Yes	Yes	Yes	Yes	Yes
Takasaki et al, 2021	Yes	Yes	Yes	Yes	Yes	Yes	Yes	Yes	Yes
Tanisaka et al, 2018	Yes	Yes	Yes	Yes	Yes	Yes	Yes	Yes	Yes
Tanisaka et al, 2020	Yes	Yes	Yes	Yes	Yes	Yes	Yes	Yes	Yes
Tanisaka et al, 2022	Yes	Yes	Yes	Yes	Yes	Yes	Yes	Yes	Yes
Tomizawa et al, 2014	Yes	Yes	Yes	Yes	Yes	Yes	Yes	No	Yes
Tomoda et al, 2021	Yes	Yes	Yes	Yes	Yes	Yes	Yes	Yes	Yes
Trindade et al. 2014	Yes	Yes	Yes	Yes	Yes	Yes	Yes	Yes	Yes
Tsou et al, 2016	Yes	Yes	Yes	Yes	Yes	Yes	Yes	Yes	Yes
Uchida et al, 2020	Yes	Yes	Yes	Yes	Yes	Yes	Yes	Yes	No
Wang et al, 2010	No	Yes	Yes	Yes	Yes	Yes	Yes	No	Yes
Wang et al, 2020	No	Yes	Yes	Yes	Yes	Yes	Yes	No	Yes
Wu et al, 2019	Yes	Yes	Yes	Yes	Yes	Yes	Yes	No	Yes
Yamada et al, 2019	Yes	Yes	Yes	Yes	Yes	Yes	Yes	Yes	Yes
Yamauchi et al, 2013	Yes	Yes	Yes	Yes	Yes	Yes	Yes	No	Yes
Zamora-Nava et al, 2020	Yes	Yes	Yes	Yes	Yes	Yes	Yes	Yes	Yes
Ali et al, 2018	Yes	Yes	Yes	Yes	Yes	Yes	Yes	Yes	Yes
Zouhairi et al, 2015	Yes	Yes	Yes	Yes	Yes	Yes	Yes	Yes	Yes
De Benito et al, 2020	Yes	Yes	Yes	Yes	Yes	Yes	Yes	No	No
Ichkhanian et al, 2020	Yes	Yes	Yes	Yes	Yes	Yes	Yes	Yes	Yes
James et al, 2019	No	Yes	Yes	Yes	Yes	Yes	Yes	Yes	Yes
Ngamruengphong et al, 2017	Yes	Yes	Yes	Yes	Yes	Yes	Yes	No	Yes
Runge et al, 2021	Yes	Yes	Yes	Yes	Yes	Yes	Yes	Yes	Yes
Abbas et al, 2018	Yes	Yes	Yes	Yes	Yes	Yes	Yes	Yes	Yes
AlMasri et al, 2022	No	Yes	Yes	Yes	Yes	Yes	Yes	Yes	Yes
Bowman et al, 2016	Yes	Yes	Yes	Yes	Yes	Yes	Yes	Yes	Yes
Falcão et al, 2012	Yes	Yes	Yes	Yes	Yes	Yes	Yes	Yes	Yes
Frederiksen et al, 2017	Yes	Yes	Yes	Yes	Yes	Yes	Yes	Yes	Yes
Habenicht et al, 2018	No	Yes	Yes	Yes	Yes	Yes	Yes	No	Yes
Koggel et al, 2021	Yes	Yes	Yes	Yes	Yes	Yes	Yes	Yes	Yes
May et al, 2019	No	Yes	Yes	Yes	Yes	Yes	Yes	No	No
Mohammad et al, 2020	No	Yes	Yes	Yes	Yes	Yes	Yes	No	Yes
Richardson et al, 2012	No	Yes	Yes	Yes	Yes	Yes	Yes	No	Yes
Saleem et al, 2012	Yes	Yes	Yes	Yes	Yes	Yes	Yes	No	Yes
Schreiner et al, 2012	Yes	Yes	Yes	Yes	Yes	Yes	Yes	Yes	Yes
Snauwaert et al, 2015	No	Yes	Yes	Yes	Yes	Yes	Yes	No	Yes
Telfah et al, 2020	No	Yes	Yes	Yes	Yes	Yes	Yes	No	Yes
Azeem et al, 2013	Yes	Yes	Yes	Yes	Yes	Yes	Yes	Yes	Yes
Mbatshi et al, 2017	No	Yes	Yes	Yes	Yes	Yes	Yes	No	No
Nennstiel et al, 2021	Yes	Yes	Yes	Yes	Yes	Yes	Yes	Yes	Yes
Wang et al, 2016	Yes	Yes	Yes	Yes	Yes	Yes	Yes	Yes	No
Sirin et al, 2020	Yes	Yes	Yes	Yes	Yes	Yes	Yes	Yes	Yes
Bukhari et al, 2018	Yes	Yes	Yes	Yes	Yes	Yes	Yes	Yes	Yes
Tonnesen et al, 2020	Yes	Yes	Yes	Yes	Yes	Yes	Yes	Yes	Yes
Wang et al, 2021	Yes	Yes	Yes	Yes	Yes	Yes	Yes	Yes	No
Choi et al, 2013	Yes	Yes	Yes	Yes	Yes	Yes	Yes	Yes	Yes

Sawas et al, 2020	Yes	Yes	Yes	Yes	Yes	Yes	Yes	Yes	Yes
Lennon et al, 2012	No	Yes	Yes	Yes	Yes	Yes	Yes	Yes	Yes
Shah et al, 2013	Yes	Yes	Yes	Yes	Yes	Yes	Yes	Yes	Yes
Kedia et al, 2019	Yes	Yes	Yes	Yes	Yes	Yes	Yes	Yes	No
Kochhar et al, 2020	Yes	Yes	Yes	Yes	Yes	Yes	Yes	Yes	No

Supplementary Table S4. Pooled outcomes and comparisons between enteroscope-assisted modalities

		Comparison (*Sig.*) DBE	SBE
	Technical success rate (95%CI)		
DBE	89.8 (87.1-92.4)		*0.65*
SBE	88.1 (85.5-90.6)		
manual SE-ERCP	70.3 (55.1-85.6)	*<0.001**	*<0.001**
	Cannulation success rate (95%CI)		
DBE	77.5 (72.4-82.6)		*0.72*
SBE	74.7 (70.7-78.6)		
manual SE-ERCP	58.8 (37.9-79.7)	*<0.001**	*<0.001**
	Therapeutic success rate (95%CI)		
DBE	71.2 (64.9-77.6)		*0.42*
SBE	69.1 (63.8-74.5)		
manual SE-ERCP	56.1 (32.0-80.2)	*<0.001**	*<0.001**
	Adverse Events rate (95%CI)		
DBE	5.7 (4.10-7.40)		*0.68*
SBE	5.2 (3.50-6.80)		
manual SE-ERCP	5.6 (-1.70-12.90)	*0.92*	*0.86*

**p<0.05 indicates statistically significant difference*

CI, confidence interval; DBE, double-balloon enteroscope-assisted ERCP; SE-ERCP, spiral enteroscope-assisted ERCP; SBE, single-balloon enteroscope-assisted ERCP.
